# Amoeboid Cells Use Protrusions for Walking, Gliding and Swimming

**DOI:** 10.1371/journal.pone.0027532

**Published:** 2011-11-09

**Authors:** Peter J. M. Van Haastert

**Affiliations:** Department of Cell Biochemistry, University of Groningen, Groningen, The Netherlands; University of Birmingham, United Kingdom

## Abstract

Amoeboid cells crawl using pseudopods, which are convex extensions of the cell surface. In many laboratory experiments, cells move on a smooth substrate, but in the wild cells may experience obstacles of other cells or dead material, or may even move in liquid. To understand how cells cope with heterogeneous environments we have investigated the pseudopod life cycle of wild type and mutant cells moving on a substrate and when suspended in liquid. We show that the same pseudopod cycle can provide three types of movement that we address as walking, gliding and swimming. In walking, the extending pseudopod will adhere firmly to the substrate, which allows cells to generate forces to bypass obstacles. Mutant cells with compromised adhesion can move much faster than wild type cells on a smooth substrate (gliding), but cannot move effectively against obstacles that provide resistance. In a liquid, when swimming, the extending pseudopods convert to side-bumps that move rapidly to the rear of the cells. Calculations suggest that these bumps provide sufficient drag force to mediate the observed forward swimming of the cell.

## Introduction

Many cells have a mode of migration known as amoeboid movement that is characterized by frequent changes in cell shape due to the extension of protrusions [1], [2]. The protrusions of an amoeboid cell, often termed pseudopods or lamellipods, are crucial for cell movement, as they determine the speed, direction and trajectory of the cell. An important aspect of cell motility is the ability of cells to respond to directional cues with oriented movement. Gradients of diffuse chemicals give rise to chemotaxis [3], [4]. Other directional cues that can induce oriented movement are temperature gradients or electric fields [5], [6]. These signals somehow modulate the direction of pseudopods such that, on average, cells move in the direction of the positional cues.

Recently, cell migration has been investigated using a ‘pseudopod-centred’ approach, in which large data sets are collected on the spatiotemporal properties of pseudopods that are extended by cells in the absence or presence of directional cues [7]–[13]. In the absence of external cues, cells are more likely to extend a new pseudopod in the direction of the existing pseudopods, and their directions are alternating to the left and right. With these characteristics cells move with strong persistence to cover a large distance in a short period [11], [14]. A shallow gradient of chemoattractant induces a small positional bias, such that cells are more likely to start a new pseudopod at the side of the cell facing the highest chemoattractant concentration [8], [9]. In addition, cells with multiple pseudopods often retract the pseudopod that is positioned in the worse direction relative to the cAMP gradient [7]. As pseudopods are generally extended perpendicular to the cell surface, this positional bias where pseudopods begin will direct the cell towards the attractant [15]. Cell migration and chemotaxis generally are studied in two dimensions as the cells crawl over various solid substrates. However, in vivo, cells move in a complex three dimensional environment [16], [17]. Such cells may experience obstacles such as other cells, soil particles, cavities, or liquids. Movement in a complex environment may require the ability to generate substantial force to resist obstacles, as well as the ability to swim [18], [19].

It has been suggested that pseudopods are self-organizing structures, which means that their organization is largely intrinsically controlled; although external signals can trigger the formation and location of a pseudopod, the pseudopod otherwise follows a typical life cycle [20]. In our studies on the movement in *Dictyostelium* cells we observed that wild type and many mutant cells extend a new pseudopod every ∼15 seconds. After ∼12 seconds, the pseudopod suddenly stops growing. In wild type cells on a solid support about 75% of the pseudopods make contact with the substrate, followed by outward expansion of the pseudopodia thereby contributing to the forward movement of the cell [9], [14]. Well after the pseudopodia cease to expand, they remain recognizable as convex extensions at the side of the cell. We have made some investigations to such “side-bumps”, because likely they are places of attachment of the cell to the substrate [21]–[24] and therefore may contribute to movement in an environment with obstacles providing enhanced resistance. Surprisingly, cells in suspension have side bumps that move to the rear of the cell, potentially providing a drag force that may contribute to the forward movement of cells in suspension. Previously it has been suggested that traveling waves of surface deformations may explain the movement of cyanobacteria [25]. Here I report on the conversion of pseudopods to side-bumps and their potential role in movement of amoeboid cells on solid supports and in suspension.

## Results and Discussion

### Walking of cells on a solid support

**Figure 1:**
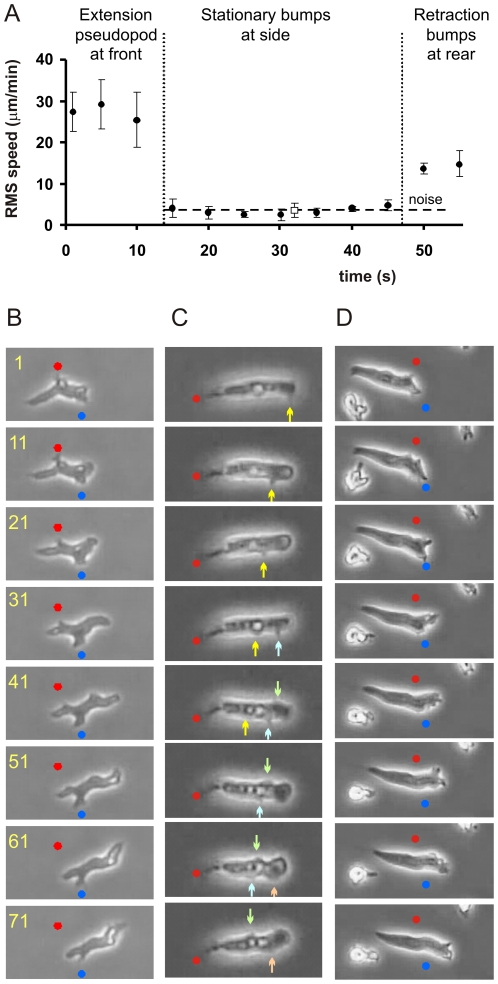
Side bumps on walking cells. A. Presented is the root-mean-square speed relative to the substrate of the tip of 20 pseudopods.. B. Images of wild-type walking cells. C. Images of tail-attached wild-type cells. D. Images of gliding *gbpD*-null cells. In the three cases the frames are static and the dots are placed at fixed positions; the arrows point to moving bumps. Numbers indicate time in seconds.

The algorithm Quimp for pseudopod analysis describes the cell boundary as a polygon of ∼100 nodes [26]. Each node has an address, and therefore its speed and convexity can be determined in subsequent images. Pseudopods are identified as extending convex areas, with the central node of this convex area assigned as the tip of the pseudopod. We followed the tip node during and after pseudopod extension. The speed of the pseudopod tip relative to the substrate during the extending phase of the pseudopod is very high (∼30 µm/min). At the end of the extension period, the speed of the tip node declines abruptly to nearly zero (Fig 1A). Interestingly, these stationary tip nodes are still present in convex areas, as identified by the computer algorithm [27] and by visual inspection of individual cells (Fig 1B). It thus appears that pseudopods frequently convert to convex bump at the side of the cell. Since the cell moves forward, the stationary bumps are found after about 1 minute in the rear of the cell, where they are retracted.

Pseudopods are formed at the front alternating to the right and left [11], [14], and therefore the sideway extensions are also alternating right/left from front to tail (Fig 1B). Previous experiments suggest that positions where pseudopods convert to side bumps are places of cell adhesion to the substrate [21]–[24]. Therefore, cell movement on a solid medium has the appearance of walking, because cells have sideway stationary “foots”; cells retract these foots in the rear while cells extend pseudopod at the front that become new foots.

### Traveling waves of convexities in tail-attached cells

*Dictyostelium* cells occasionally appear to be attached to the substrate exclusively at the tail region, whereas other parts of the cell move freely in suspension (Fig. 1C). This interpretation is based on the observations that i) the tail touches the surface in the focal plane of the tail ii) the position of the tail does not change (thus the tail does not move), and iii) the rest of the cell can move; sometimes the cell is relatively stationary as in figure 1C (and therefore pseudopod and bump data can be easily collected for a few minutes) and sometimes cells move actively to the right and left (“waving”). In wild type cells, about 5–10% of the cells show this behavior. In some mutants, such as talinB-null cells, tail-attached movement is very common (83% of the cells). Tail-attached cells extend pseudopods at nearly the same frequency compared to surface-attached cells (Table S1). However, pseudopods grow for a shorter period and are also smaller. As in cell body-attached cells, the convex pseudopod tips frequently convert to side bumps. Interestingly, these side bumps travel to the rear of the cell (Fig. 1C) at a speed of about -13 µm/min; the minus sign is to indicate that side-bumps travel in a direction opposite to extending pseudopods. After approximately 1 minute the side bumps have arrived at the tail of the cell.

### Swimming cells use pseudopods that convert to paddles

**Figure 2:**
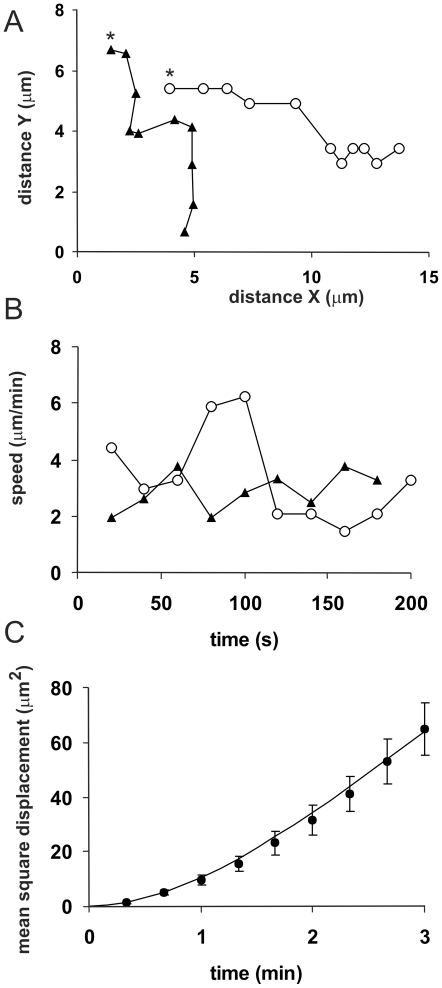
Swimming cells. The trajectory of 8 tail-attached cells was followed after detachment from the surface. A. Tracks of 2 swimming cells at 20 s interval; * indicate the start. B. Speed of the same 2 cells. C. Mean square displacement 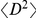 of 8 swimming cells. The parameters of the equation for a persistent random walk in three dimensions 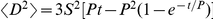 were fitted to the data points; the line presents the optimal fit with speed S = 3 µm/min and persistence time P = 1.3 min.

Occasionally the tail-attached cells detach from the substrate. Although such cells will slowly sink, they can be followed while completely free in suspension during a few minutes (Fig. 2A). We followed 8 tail-attached cells after they detach from the substrate. Such cells continue to form pseudopods and side bumps that travel to the rear of the cell, with essentially the same properties as tail-attached cells. The swimming cells move forward (i.e. in the direction of the extended pseudopods) at a slow speed of ∼ 3 µm/min (Table S1). The trajectories of swimming cells reveal persistent directional movement (Fig. 2C).

### Gliding of adhesion-defective mutant cells on a smooth surface

**Figure 3:**
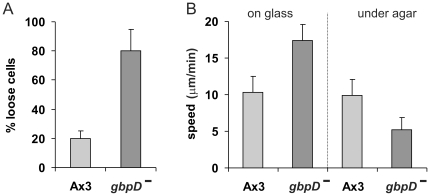
Gliding cells. A. Adhesion of wild type and *gbpD*-null cells, expressed as the fraction of cells that detach from a plastic surface after shaking in buffer for 1 hour [28]. B. Speed of wild-type and mutant cells on a solid support and under a block of agar.

The GTP-binding protein Rap1 has been shown to be involved in cell adhesion [28]. Cells lacking GbpD, a Rap-GEF, exhibit strongly reduced adhesion (Fig 3A). We analyzed pseudopod formation in *gbpD*-null cells (Table S1). Pseudopods are formed at a frequency and speed that are similar compared to wild type cells, but they grow longer and are therefore larger. After the pseudopod tip stops outward expansion, the pseudopod rarely continues as convex extension at the side of the cell body (Fig.1D). Instead, the cytosol flows into the pseudopod and the old pseudopod tip merges with the cell body. Interestingly, these cells have enhanced speed of movement, 17 µm/min, compared to ∼10 µm/min for wild type cells. The enhanced speed was also observed for other mutants with reduced adhesion: *talinA*-null cells with defects in the cytoskeleton [29] and *sadA*-null cells with defects in adhesion molecules [30]. The adhesion-defective mutant cells move nearly with the speed of the forward moving pseudopods, suggesting that cells in the absence of strong adhesion glide over the substrate.

Can cells with reduced adhesion move against obstacles? Wild-type and *gbpD*-null cells were covered with a block of agar to provide resistance. Whereas movement of wild type cells under agar is only slightly slower than movement on agar, the speed of *gbpD*-null cells is strongly reduced from ∼17 µm/min to ∼5 µm/min under agar.

### How fast can cells glide, walk and swim?

The maximal speed of walking or gliding cells on a substrate depends on the frequency *F* and size *l_p_* of extending pseudopods according to 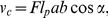
(1)where *a*  =  fraction of pseudopods that contribute to movement, *b*  =  positional overlap of pseudopods, and *α*  =  the mean angle between pseudopod and forward cell movement. The experimental data (Table S2) predict that wild type cells can walk at a maximal speed of 11.4 µm/min, which is close to the observed speed of 10.4 µm/min.

Gliding *gbpD*-null cells extend pseudopods at nearly the same frequency as wild type cells, but with some subtle differences: pseudopods are larger and rarely retracted, thereby providing more forward movement to the cells. Using these measured pseudopod properties, the predicted speed of gliding cells is 17.8 µm/min, which is close to the observed speed of 17.3 µm/min.

Apparently, the lower speed of wild type cells is attributed to the shorter pseudopods and the more frequent retraction of new pseudopods. Why? Movement of substrate-attached cells can be regarded as the transport of material from behind the attachment zone at the rear of the cell to before the attachment zone at the front of the cell. Materials to be transported are cytoplasm with organelles and plasma membrane. Cytoplasm may flow freely to the front by hydrostatic pressure [31], but transport of membrane may be restricted by adhesion of the cell to the surface. The reduced flow of membrane may lead to increased membrane tension at the front of the cell, which could impair pseudopod growth and induce retraction of newly formed pseudopods, as is observed in wild type cells.

How fast can cells swim in liquid? An object moving in a fluid will experience a drag force that, according to Newton's third law, will induce an equal counterforce on the object. For small objects the drag occurs at low Reynolds numbers [32] and therefore the drag force *F_D_* is given by the Stokes equation: 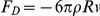, where *ρ* is the viscosity of the fluid, *R* is the radius of the frontal cross sectional area of the object and *v* is the speed of the object. A swimming *Dictyostelium* cell may experience three forces: the drag induced by the extending pseudopod, the drag induced by the rearward moving bumps, and the drag induced by the total movement of the cell. Assuming that these three drag forces experience the same viscosity, the balanced force equation yields: 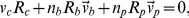
(2)where the subscripts indicate cells (c) bumps (b) or pseudopods (p), 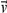 indicates the mean speed of the object in the direction of the cell and *n* indicates the mean number of moving bumps or pseudopods. We measured these properties for cells in suspension (Table S2), predicting an average swimming rate of ∼ 3.5 µm/min, which is close to the observed rate of 3 µm/min for freely swimming *Dictyostelium* cells.

### Conclusions

**Figure 4:**
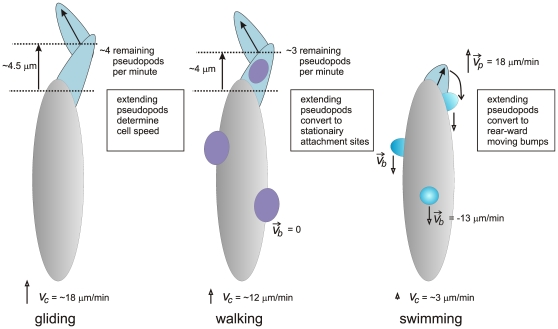
Model of gliding, walking and swimming. All cells extend pseudopods at a frequency of ∼4 per minute. In gliding cells, pseudopods are large and all contribute to forward moving; side bumps are rare. In walking cells, pseudopods are smaller and only ∼75% of pseudopods contribute to forward movement; these pseudopods convert to side-bumps that are stationary relative to the substrate. In swimming cells, pseudopods are small and convert to side-bumps that move to the rear of the cell. On average walking and swimming cells have ∼3 side-bumps (see table S1)

Polarized amoeboid cells may move by three modes, walking, gliding and swimming (Fig.4). Investigations to swimming cells are complicated because cells in suspension will sink. In isodense suspensions of ficol cells can be observed easily [18], but ficol may induce an osmotic response or it may provide some form of structure to move on, as ficol may not be a Newtonian solution [33]. We investigated cells that detached from the surface by which they move in suspension, and obtained a swimming speed of ∼3 µm/min, about 4-times slower that the crawling speed of ∼12 µm/min. In ficol, cells swim and crawl at about the same speed of 4.2 and 3.8 µm/min, respectively [18]. The current observations suggest that the three moving modes, walking, gliding and swimming, are all based on pseudopods, which are extending convex areas of the cell boundary. At the end of the extension period the convex pseudopods often convert to convex bumps at the side of the cell, which move in about 1 minute to the rear of cells in suspension. The ∼3 rearward moving bumps provide sufficient drag force to explain the observed forward movement of swimming cell. Pseudopods of cells attached to a substrate also convert to side bumps that end up in the rear of the cell after about 1 minute. However, these bumps do not move, but are fixed to the substrate, presumably because they form attachment sides of the cell to the substrate [21]–[24]. Surprisingly, adhesion-defective mutant cells move at a much higher speed than wild type cells on a smooth surface, but exhibit a much lower speed then wild type cells when exposed to strong resistance, such as movement under a block of agar. *Dictyostelium* cells live in a heterogeneous environment composed of soil particles and surrounding liquid. Cells move probably most of the time on 2D surfaces of soil particles, but may also experience clefts and obstacles. Cells have the ability to walk on these complicated surfaces with stronger adhesion, and to swim in water, all using essentially the same cycle of pseudopod formation with conversion to sideway extensions. This may allow the cells to effectively move optimally in its physically complex habitat.

## Methods

The strains used are wild type AX3 and *gbpD*-null cells lacking gene encoding a Rap-GEF [28]. Cells were grown in HG5 medium (contains per liter: 14.3 g oxoid peptone, 7.15 g bacto yeast extract, 1.36 g Na_2_HPO_4_⋅12H_2_O, 0.49 g KH_2_PO_4_, 10.0 g glucose), harvested in PB (10 mM KH2PO4/Na2HPO4, pH 6.5), and allowed to develop in 1 ml PB for 5 hours in a well of a 6-wells plate (Nunc). Movies of starved cells in PB on an objective glass were recorded with an inverted light microscope (Olympus Type CK40 with 20x objective) and images were captured at a rate of 1 frame/second with a JVC CCD camera. For movement under agar, cells were covered with an approximately cubic (length 3 mm) block of 1.5% agar in PB. Excess buffer was removed and movies were recorded as described above.

Images were analyzed with the fully automatic algorithm Quimp3 [26]. In short, the program uses an active contour analysis to identify the outline of the cell as ∼150 nodes [34]. The local curvature of the cell outline is defined as the angle of the line segments pointing from a given node to its two neighbors, and the program identifies the central node of convex regions. With the convexity and area change of the nodes, extending pseudopodia were identified that fulfill the requirement of used-defined minimal number of adjacent convex nodes and minimal area change. The x,y coordinates of the central convex node of the convex area were recorded from start to end of pseudopod growth as described [26]; the position of this node was followed till the node disappeared by retraction.

The data are presented as the means and standard deviation (SD), where *n* represents the number of pseudopodia or number of cells analyzed, as indicated. The number of bumps in swimming and tail-attached cells is based on the observed number bumps at the lateral and upper sides of the cell, and multiplied by 4/3 to account for the invisible bumps in the lower side of the cell.

## Supporting Information

Table S1 Properties of pseudopods, bumps and cells for three modes of movement.
(DOC)

Table S2 Three modes of pseudopod-based movement
(DOC)

## References

[1] Friedl P, Wolf K (2009). Plasticity of cell migration: a multiscale tuning model.. J Cell Biol.

[2] Lammermann T, Sixt M (2009). Mechanical modes of 'amoeboid' cell migration.. Curr Opin Cell Biol.

[3] Weiner OD (2002). Regulation of cell polarity during eukaryotic chemotaxis: the chemotactic compass.. Curr Opin Cell Biol.

[4] Hoeller O, Kay R (2007). Chemotaxis in the absence of PIP3 gradients.. Current Biology.

[5] Bahat A, Eisenbach M (2006). Sperm thermotaxis.. Mol Cell Endocrinol.

[6] Zhao M (2009). Electrical fields in wound healing-An overriding signal that directs cell migration.. Semin Cell Dev Biol.

[7] Andrew N, Insall RH (2007). Chemotaxis in shallow gradients is mediated independently of PtdIns 3-kinase by biased choices between random protrusions.. Nat Cell Biol.

[8] Arrieumerlou C, Meyer T (2005). A local coupling model and compass parameter for eukaryotic chemotaxis.. Dev Cell.

[9] Bosgraaf L, Van Haastert PJM (2009). Navigation of chemotactic cells by parallel signaling to pseudopod persistence and orientation.. PLoS One.

[10] Maeda YT, Inose J, Matsuo MY, Iwaya S, Sano M (2008). Ordered patterns of cell shape and orientational correlation during spontaneous cell migration.. PLoS ONE.

[11] Li L, Norrelykke SF, Cox EC (2008). Persistent cell motion in the absence of external signals: a search strategy for eukaryotic cells.. PLoS ONE.

[12] Takagi H, Sato MJ, Yanagida T, Ueda M (2008). Functional analysis of spontaneous cell movement under different physiological conditions.. PLoS ONE.

[13] Insall RH (2010). Understanding eukaryotic chemotaxis: a pseudopod-centred view.. Nat Rev Mol Cell Biol.

[14] Bosgraaf L, van Haastert PJM (2009). The Ordered Extension of Pseudopodia by Amoeboid Cell in the Absence of external Cues.. PLoS ONE.

[15] Van Haastert PJ, Bosgraaf L (2009). The local cell curvature guides pseudopodia towards chemoattractants.. Hfsp J.

[16] Franck C, Maskarinec SA, Tirrell DA, Ravichandran G Three-dimensional traction force microscopy: a new tool for quantifying cell-matrix interactions. PLoS One.

[17] Provenzano PP, Eliceiri KW, Keely PJ (2009). Shining new light on 3D cell motility and the metastatic process.. Trends Cell Biol.

[18] Barry NP, Bretscher MS (2010). Dictyostelium amoebae and neutrophils can swim.. Proc Natl Acad Sci U S A.

[19] Bae AJ, Bodenschatz E (2010). On the swimming of Dictyostelium amoebae.. Proc Natl Acad Sci U S A.

[20] Karsenti E (2008). Self-organization in cell biology: a brief history.. Nat Rev Mol Cell Biol.

[21] Weber I (2006). Is there a pilot in a pseudopod?. Eur J Cell Biol.

[22] Uchida KS, Yumura S (2004). Dynamics of novel feet of Dictyostelium cells during migration.. J Cell Sci.

[23] Fukui Y, Inoue S (1997). Amoeboid movement anchored by eupodia, new actin-rich knobby feet in Dictyostelium.. Cell Motil Cytoskeleton.

[24] Iwadate Y, Yumura S (2008). Actin-based propulsive forces and myosin-II-based contractile forces in migrating Dictyostelium cells.. J Cell Sci.

[25] Stone H, Samuel A (1996). Propulsion of Microorganisms by Surface Distortions.. Phys Rev Lett.

[26] Bosgraaf L, Van Haastert PJM (2009). Quimp3, an automated pseudopod-tracking algorithm.. Cell Adh Migr.

[27] Bosgraaf L, van Haastert PJ, Bretschneider T (2009). Analysis of cell movement by simultaneous quantification of local membrane displacement and fluorescent intensities using Quimp2.. Cell Motil Cytoskeleton.

[28] Kortholt A, Rehmann H, Kae H, Bosgraaf L, Keizer-Gunnink I (2006). Characterization of the GbpD-activated Rap1 pathway regulating adhesion and cell polarity in Dictyostelium discoideum.. J Biol Chem.

[29] Niewohner J, Weber I, Maniak M, Muller-Taubenberger A, Gerisch G (1997). Talin-null cells of Dictyostelium are strongly defective in adhesion to particle and substrate surfaces and slightly impaired in cytokinesis.. J Cell Biol.

[30] Fey P, Stephens S, Titus MA, Chisholm RL (2002). SadA, a novel adhesion receptor in Dictyostelium.. J Cell Biol.

[31] Keren K, Yam PT, Kinkhabwala A, Mogilner A, Theriot JA (2009). Intracellular fluid flow in rapidly moving cells.. Nat Cell Biol.

[32] Dusenbery D (2009). Living at Micro Scale: The Unexpected Physics of Being Small Harvard University Press, Cambridge,.

[33] Winet H (1976). Ciliary propulsion of objects in tubes: wall drag on swimming Tetrahymena (Ciliata) in the presence of mucin and other long-chain polymers.. J Exp Biol.

[34] Bosgraaf L, Van Haastert PJM, Bretschneider T (2009). Analysis of cell movement by simultaneous quantification of local membrane displacement and fluorescent intensities using Quimp2.. Cell Motil Cytoskel.

