# Supplementary material for: Amoeboid Cells Use Protrusions for Walking, Gliding and Swimming
Source: PLoS One. 2011 Nov 9;6(11):e27532. doi: 10.1371/journal.pone.0027532 (PMC3212573; doi:10.1371/journal.pone.0027532)
Supplement: Table S1 — Properties of pseudopods, bumps and cells for three modes of movement. (DOC) [file pone.0027532.s001.doc]

|  |  | type of movement | | | | | |
| --- | --- | --- | --- | --- | --- | --- | --- |
| Property | Symbol | Substrate attached | | Tail attached | | Gliding | |
|  | (units) | Mean | SD | mean | SD | mean | SD |
| ***pseudopods*** |  | **n=335** |  | **n=135** |  | **n=191** |  |
| size | *l_p_* (µm) | 5.78 | 2.35 | 2.69 | 0.67 | 6.40 | 0.63 |
| growth time | (s) | 12.23 | 1.17 | 7.57 | 2.23 | 21.51 | 3.51 |
| growth speed | *v_p_* (µm/min) | 28.36 | 3.73 | 21.34 | 4.12 | 19.59 | 1.82 |
| Frequency | *F* (1/min) | 3.87 | 0.45 | 3.63 | 1.30 | 4.11 | 0.31 |
| mean number | *n_p_* | 0.82 | 0.11 | 0.46 | 0.19 | 1.47 | 0.18 |
| angle relative to direction cell | *α* (degrees) | 28 | 14 | 40 | 7 | 25 | 7 |
| spatial overlap previous | *a* | 0.77 | 0.07 | 1.00 |  | 0.81 | 0.05 |
| fraction maintained | *b* | 0.75 | 0.15 | 1.00 |  | 0.92 | 0.05 |
|  |  |  |  |  |  |  |  |
| ***bumps*** |  | **n=273** |  | **n=107** |  |  |  |
| pseudopods converting to bumps | (%) | 82 |  | 80 |  | <10 |  |
| size (extension) | (µm) | 1.76 | 0.46 | 1.78 | 0.45 |  |  |
| size (radius) | (µm) | 0.92 | 0.28 | 0.95 | 0.26 |  |  |
| mean number | (*n_b_*) | 2.73 | 0.47 | 3.08 | 0.53 |  |  |
| life time | (min) | 0.86 | 0.13 | 1.07 | 0.22 |  |  |
| speed relative to substrate | *v_b_* (µm/min) | -0.04 | 1.34 | -13.18 | 2.69 |  |  |
|  |  |  |  |  |  |  |  |
| **cells** |  | **n=27** |  | **n=22** |  | **n=24** |  |
| size (length) | (µm) | 17.62 | 2.46 | 25.05 | 4.01 | 26.74 | 2.13 |
| size (radius) | (µm) | 3.84 | 0.36 | 2.76 | 0.24 | 2.95 | 0.42 |
| speed relative to substrate | *v_c_* (µm/min) | 10.36 | 2.17 | 0.10 | 1.39 | 17.34 | 2.25 |
| swimming speed (n=8) | *v_c_* (µm/min) |  |  | 2.98 | 1.30 |  |  |

**Table S1. Properties of pseudopods, bumps and cells for three modes of movement.**

Analyzed were the indicated number of cells, providing information on the indicated number of extending pseudopods or convex areas at the side of the cell (bumps). The swimming speed is the speed of tail-attached cells after they detach from the surface.
