# Supplementary material for: Amoeboid Cells Use Protrusions for Walking, Gliding and Swimming
Source: PLoS One. 2011 Nov 9;6(11):e27532. doi: 10.1371/journal.pone.0027532 (PMC3212573; doi:10.1371/journal.pone.0027532)
Supplement: Table S2 — Three modes of pseudopod-based movement (DOC) [file pone.0027532.s002.doc]

**Table S2. Three modes of pseudopod-based movement**

| **Mode of movement** | Speed (µm/min) | |
| --- | --- | --- |
|  | Calculated | Observed |
| Gliding = Movement of adhesion defective gbpD-null cells on substrate$v_{c}=Fl_{p}ab\cos\alpha$ (1) F = pseudopod frequency = 4.11 ± 0.31  *l_p_* = length pseudopod = 6.40 ± 0.63  *a* = fraction of pseudopods contributing to movement = 0.92 ± 0.05  *b* = spatial overlap pseudopods = 0.81 ± 0.05  *α* = angle between pseudopod and direction of cell = 25 ± 7 degrees | 17.8 | 17.3 ± 2.3 |
| Walking = Movement of wild-type cells on substrate$v_{c}=Fl_{p}ab\cos\alpha$ (1) F = pseudopod frequency = 3.87 ± 0.45  *l_p_* = length pseudopod = 5.78 ± 2.35  *a* = fraction of pseudopods contributing to movement = 0.75 ± 0.15  *b* = spatial overlap pseudopods = 0.77 ± 0.07  *α* = angle between pseudopod and direction of cell = 28 ± 14 degrees | 11.4 | 10.4 ± 2.2 |
| Swimming = Movement of wild-type cells in suspension(2) $\vec{v}_{b}$ = mean speed of bumps in the direction of the cell, relative to medium  $\vec{v}_{b}=v_{b}+v_{c}$; $v_{b}$= -13.2 ± 2.7 µm/min $\vec{v}_{p}$ = mean speed of pseudopods in the direction of the cell, relative to medium  $\vec{v}_{p}=\cos(\alpha)(v_{p}+v_{c})$; $\alpha$ = 40 ± 7 degrees; $v_{p}$ = 21 ± 4 µm/min $R_{b}$ = radius of mean frontal cross sectional area of bump = 0.92 ± 0.3 µm $R_{p}$ = radius of mean frontal cross sectional area of pseudopod = 1.2 ± 0.3 µm $R_{c}$ = radius of mean frontal cross sectional area of cell = 5.0 ± 1 µm $n_{b}$ = mean number of bumps per cell = 3.1 ± 0.5 $n_{p}$ = mean number of moving pseudopods per cell = 0.46 ± 0.19 | 3.45 | 3.0 ± 1.3 |
